# Visuo-motor coordination ability predicts performance with brain-computer interfaces controlled by modulation of sensorimotor rhythms (SMR)

**DOI:** 10.3389/fnhum.2014.00574

**Published:** 2014-08-06

**Authors:** Eva M. Hammer, Tobias Kaufmann, Sonja C. Kleih, Benjamin Blankertz, Andrea Kübler

**Affiliations:** ^1^Department of Psychology I, University of WürzburgWürzburg, Germany; ^2^Institute of Clinical Medicine, University of OsloOslo, Norway; ^3^Neurotechnology Group, Berlin Institute of TechnologyBerlin, Germany; ^4^Bernstein Focus: Neurotechnology (BFNT)Berlin, Germany

**Keywords:** brain-computer interfaces, sensorimotor rhythms, predictors, visuo-motor coordination abilities, attentional impulsivity

## Abstract

Modulation of sensorimotor rhythms (SMR) was suggested as a control signal for brain-computer interfaces (BCI). Yet, there is a population of users estimated between 10 to 50% not able to achieve reliable control and only about 20% of users achieve high (80–100%) performance. Predicting performance prior to BCI use would facilitate selection of the most feasible system for an individual, thus constitute a practical benefit for the user, and increase our knowledge about the correlates of BCI control. In a recent study, we predicted SMR-BCI performance from psychological variables that were assessed prior to the BCI sessions and BCI control was supported with machine-learning techniques. We described two significant psychological predictors, namely the visuo-motor coordination ability and the ability to concentrate on the task. The purpose of the current study was to replicate these results thereby validating these predictors within a neurofeedback based SMR-BCI that involved no machine learning.Thirty-three healthy BCI novices participated in a calibration session and three further neurofeedback training sessions. Two variables were related with mean SMR-BCI performance: (1) a measure for the accuracy of fine motor skills, i.e., a trade for a person’s visuo-motor control ability; and (2) subject’s “attentional impulsivity”. In a linear regression they accounted for almost 20% in variance of SMR-BCI performance, but predictor (1) failed significance. Nevertheless, on the basis of our prior regression model for sensorimotor control ability we could predict current SMR-BCI performance with an average prediction error of *M* = 12.07%. In more than 50% of the participants, the prediction error was smaller than 10%. Hence, psychological variables played a moderate role in predicting SMR-BCI performance in a neurofeedback approach that involved no machine learning. Future studies are needed to further consolidate (or reject) the present predictors.

## Introduction

Prediction of behavior, performance or decisions of individuals or groups is a popular theme in modern psychology research. One or more predictor variables are acquired and used to predict an ensuing state. Recently, developments of predictors have been introduced to Brain-Computer Interface research.

Brain-Computer Interfaces (BCI) are direct connections between the brain and a computer (for review, e.g., Kübler et al., 2001; Birbaumer and Cohen, 2007; Kübler and Müller, 2007; Wolpaw and Wolpaw, 2012). They translate brain signals into operational commands for technical devices. Thus, patients with severe motor impairment are able to communicate with their environment without use of their natural, motor dependent communication channels. Apart from communication, BCIs have proved potentially valuable for environmental control, such as computer based applications, wheelchairs or arm prosthesis (e.g., Millán et al., 2010). Non-invasive BCIs rely on modulation of sensorimotor rhythms (SMR; Pfurtscheller and Neuper, 1997; Pfurtscheller et al., 1997; for a recent review, Pfurtscheller and McFarland, 2012), event related potentials such as the P300 (Farwell and Donchin, 1988; for a review, Kleih et al., 2011; Sellers et al., 2012) or on steady state visually evoked potentials (SSVEP, Middendorf et al., 2000; for a recent review, Allison et al., 2012).

BCIs controlled by motor imagery are based on modulation of SMR, i.e., rhythms in the frequency range of alpha (8–13 Hz) and beta (20–30 Hz) bands recorded from sensorimotor areas. The SMR desynchronizes (event-related desychronization; ERD) with movement, movement preparation or movement imagery (MI). Thus, SMR modulated by MI allows for muscle-independent BCI control. We will further refer to this BCI as SMR-BCI.

### Inefficacy in SMR-BCI

To control an SMR-BCI application effectively, an accuracy of at least 70% as criterion level is required (Kübler et al., 2001). Unfortunately, often this criterion level cannot be reached by 10–50% of motor impaired or healthy end-users (Guger et al., 2003; Blankertz et al., 2010). In previous reports these participants were called “BCI illiterates” (Kübler and Müller, 2007), now we recommend the term “BCI inefficiency” (Kübler et al., 2011) because the former label could be perceived as derogatory against BCI users. Currently, there is only little knowledge about the “BCI-inefficiency” phenomenon and the determinants of learning how to control a BCI. Hence, establishing reliable and valid SMR-BCI predictors may contribute to a better understanding of how the brain instantiates BCI control and, more application oriented, may help to better adapt BCI to individual users.

### SMR-BCI predictors

In a longitudinal study of Nijboer et al. (2010), six severely impaired patients with amyotrophic lateral sclerosis (ALS) were trained with an SMR-BCI for 20 sessions. The authors’ intention was to investigate the influence of disease severity, quality of life, severity of depressive symptoms, motivation to control a BCI and current mood on BCI performance. Motivational factors were related to SMR-BCI performance, mood was not. In particular, challenge and mastery confidence were positively related, incompetence fear was negatively related to SMR-BCI performance. Kleih et al. (2013) also demonstrated in samples with healthy BCI users and stroke patients that factors of motivation were related to SMR-BCI performance. A negative correlation between SMR-BCI performance and incompetence fear and the fear to fail was a consistent result in both samples. In healthy subjects a positive correlation was found between SMR-BCI performance and “locus of control when dealing with technology” (Burde and Blankertz, 2006). However, recently, Witte et al. (2013) reported that a high score of “locus of control by dealing with technology” was negatively correlated with power of SMR. They concluded that subjects with lower control beliefs could control the SMR-BCI more effectively because they were more relaxed during the neurofeedback training. These divergent results indicate that replication and validation studies for predictors and correlates of BCI performance are urgently needed.

Further, in healthy participants, performance was superior when instructed to imagine movement “kinesthetically” (Neuper et al., 2005) and when they were in better mood and had more “mastery confidence”, another component of motivation (Nijboer et al., 2008). Halder et al. (2011) investigated differences in brain activation patterns of good performers compared to low performers and found significant differences in prefrontal (DLPFC) as well as supplementary and premotor areas, i.e., high aptitude users displayed significantly higher task-related activation in these areas.

### Previous study to investigate predictors

In an extensive bi-center study, we investigated whether psychological and physiological parameters would predict SMR-BCI performance based on the Berlin Brain-Computer Interface (BBCI; Blankertz et al., 2010; Hammer et al., 2012), a so called machine learning approach that provides BCI control during the first session after a 30 min calibration period (Blankertz et al., 2007). Since those results serve as a basis for the current study, we present a summery about the methods, results and implications:

Eighty healthy participants performed a motor imagery task, first during calibration and subsequently in three feedback sessions, during which they had to operate a one-dimensional (1D) cursor. Blankertz et al. (2010) proposed a neurophysiological predictor of BCI performance which was determined from a 2 min recording of a “relax with eyes open” condition using two Laplacian EEG channels. The neurophysiological predictor accounted for 28% of the variance in SMR BCI performance (Blankertz et al., 2010).

Psychological parameters were collected with an electronic test-battery including a substantial number of clinical, personality and performance tests (Hammer et al., 2012). Two variables significantly predicted SMR feedback performance: “overall mean error duration”, an output variable of the Two-Hand Coordination Test (*r* = 0.42; 2HAND; Schuhfried, 2007a) accounted for 11% of the variance in BCI performance and “performance level”, an output variable of the Attitudes Towards Work test (*r* = 0.50; AHA, Kubinger and Ebenhöh, 1996), accounted for 19% of the variance.

In Hammer et al. (2012) we argued that the small number of significant psychological predictors was owed to the machine learning approach to BCI control that relies mainly on pattern recognition, and less on human learning.

### Aims of the current study

Firstly, we were aiming at replicating and thereby consolidating the two psychological predictors previously found. Secondly, we were interested whether we would find more psychological predictors when applying a neurofeedback approach with individual feature selection, but without adaptation (machine learning), in which an increase of performance with training can solely be ascribed to human learning.

We hypothesized that “overall mean error duration” (2HAND) and “performance level” (AHA) would also, or even better, predict performance in the neurofeedback approach to SMR-BCI control, because both approaches (neurofeedback and machine learning) require coordination between visual input and motor imagery and attention. Provided true, we aimed at predicting the current SMR-BCI performance on the basis of the regression models described by Hammer et al. (2012), which would consolidate the validity of the predictors. Additionally, we explored whether further psychological variables from the test-battery compiled by Hammer et al. (2012) would predict SMR-BCI based neurofeedback results.

## Materials and methods

The study was conducted at the University of Würzburg, Institute of Psychology, Department of Psychology I, approved by the Ethical Review Board of the Medical Faculty, University of Tübingen and in accordance with the World Medical Association (2013). All participants signed informed consent and either received 8€/h or course credits.

### Participants

*N* = 33 healthy participants with *no* previous experience in SMR-BCI took part in the study, i.e., none of the participants were included in the previous study (Hammer et al., 2012). Most participants were students. Due to equipment failure, data from one participant was excluded. The final sample comprised *N* = 32 participants (18 female, 14 male), mean age was 24.20 years (*SD* = 2.88; range 19.67–32.41).

### Experimental paradigms

**Figure 1:**
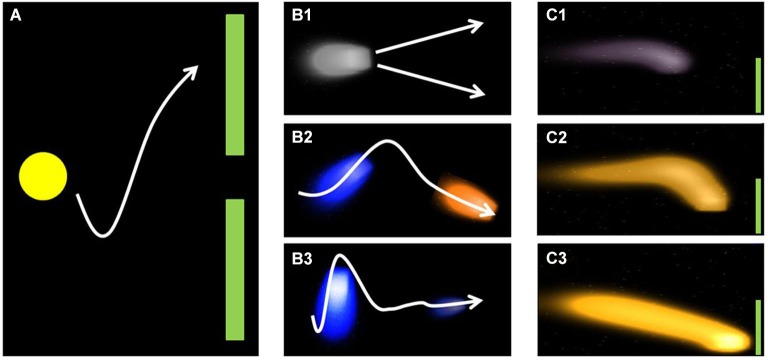
**Illustration of classic (A) and fluid (B+C) feedback approach.** Figure reproduced from Kaufmann et al. (2011) with permission of the International Journal of Bioelectromagnetism.

Two different feedback paradigms were used to feedback SMR modulation to the participants. Around half of the participants (*n* = 19) were confronted with the classic cursor task paradigm as implemented in BCI2000 (Schalk et al., 2004; see Figure 1A). At the beginning of each trial, a target is displayed on the right top or bottom of the monitor. A ball (cursor) is positioned at the center of the left margin of the screen and throughout the trial moves with continuous speed from the left to the right. Participants control the vertical movement of the cursor by modulating their SMR. Each trial is of same duration and successful if the presented target (either bottom or top) is hit. Cursor movement is based on the integrated classifier output, i.e., it is based on classification of the current trial. For example if the cursor is at top and foot imagery is classified the cursor would start moving downward, yet the actual position would not relate to current foot classification. Consequently, position of the cursor tells the participant which class has mostly been classified throughout the trial. Thus, only the observation of cursor movement (not the current position) provides information about the currently classified MI.

This entails the problem that participants cannot interfere before the actual direction change is visible. We addressed this issue by providing the other half of participants (*n* = 14) with a new feedback paradigm that aimed at feeding back more information to the participants than the classic cursor task paradigm. Firstly, color of the cursor changed according to the MI currently classified (orange = foot; blue = hand, Figure 1B2). Secondly, instead of a stable ball shape, the cursor was displayed as a fluid that increased or decreased according to the classification certainty (Figure 1B3, C1–C3). Thirdly, the fluid changed its position according to the integrated classifier output of the current trial (Figure 1B3). Similar to the classic cursor task paradigm, participants’ task was to steer the fluid toward the target (Kaufmann et al., 2011), that is displayed on the right top or bottom of the monitor (Figure 1B1). As groups did not differ in their performance (3 × 2 repeated measures ANOVA, see data analysis) we merged the data for the analysis of psychological predictors. The SMR-BCI performance as a function of paradigms is discussed elsewhere (Kaufmann et al., 2011).

### Experimental schedule

All participants attended one session for assessment of the psychological test battery prior to BCI training (see Section Psychological Test Battery). BCI training started with a calibration session comprising three runs. Three classes MI were performed during the calibration sessions: MI of the right hand, left hand and both feet. Each MI was performed 25 times and the order of trials was randomized. Participants were instructed to imagine the movement kinesthetically (Neuper et al., 2005).

Electrode positions and corresponding frequency bands, that provided the best discrimination were computed for each combination of classes. The highest achieved discrimination coefficient determined the combination of classes for the feedback sessions (e.g., left vs. right MI).

Three training sessions were performed. The first session was on the same day following calibration, whereas sessions two and three were scheduled on two separate days within 1 week. Before and after each feedback session, mood and motivation were assessed with visual analog scales.

### Psychological test battery

Following Hammer et al. (2012) the psychological test-battery comprised three groups of tests: performance tests, personality tests and clinical tests. Three of those were only available as paper-pencil tests, the others were presented electronically by the “Vienna Test System” (VTS), a computerized psychological assessment tool (SCHUHFRIED GmbH). Psychological testing lasted about 2 h. All tests are listed below, for an extensive description of the tests and their variables see Hammer et al. (2012). Because of their special pertinence for the current study, the 2HAND and the AHA are described more detailed, likewise the *Barrat Impulsiveness Scale—BIS-15* (Spinella, 2007) which was added to the test-battery.

#### Performance tests

*Two-Hand Coordination—2HAND* (Schuhfried, 2007b): the test focuses on sensorimotor coordination between eye and hand and coordination between left and right hand. The test measures speed and coordination accuracy in fine and small-scaled movements. The task is to move a visually displayed, small red dot along a pre-set track that is presented on a screen. Participants control the dot with two joysticks or knobs, one for each dimension (horizontal/vertical). If the dot leaves the track, an audible signal reminds subjects to stay on track. The outcome variable “mean error duration” refers to the time when the dot was out of the line—averaged across all runs. Therefore it is an indicator of fine motor skills and the exactness of information processing (to detect small deviations and to counteract with compensatory movements).

*Cognitrone—COG* (Schuhfried, 2007a): the COG is a general performance test for the assessment of attention and concentration. Participants had to indicate to which of four, a geometrical figure was congruent.

*Verbal Learning Test—VLT* (Sturm and Willmes, 1994b): the VLT assesses verbal learning abilities by presenting neologisms. The participants were instructed that 160 words would be presented and that they would have to memorize them because some words would be recurring during the tests.

*Non-verbal Learning Test—NVLT* (Sturm and Willmes, 1994a): the NVLT assesses non-verbal learning processes by presenting graphical material that is difficult to verbalize. This test thus allows for detection of material (verbal, spatial) specific learning disorders in comparison with the VLT. Procedure and outcome variables were the same as in the VLT.

#### Personality tests

*Big Five Plus One Personality-Inventory—B5PO* (Holocher-Ertl et al., 2003): the B5PO is a self-report measure and comprises the six dimensions “empathy”, “emotional stability”, “extraversion”, “conscientiousness”, “openness to experience” and “agreeableness”.

*Fragebogen zu Kontrollüberzeugungen* (locus of control)—*IPC-Scales* (Krampen, 1981): this test assesses locus of control and comprises three scales: “Internal scale” (I), “powerful others scale” (P) and “chance scale” (C).

*Attitudes towards work—AHA* (Kubinger and Ebenhöh, 1996): the AHA is an objective personality test which assesses “exactitude”, “decisiveness”, “impulsivity/reflexivity”, “aspiration level”, “performance level”, “frustration tolerance”, “target discrepancy” and “performance motivation”. The AHA comprises three subtests. In the subtest “encode symbols”, participants are supposed to assign symbols to abstract shapes according to a pre-set code, and are asked to estimate their performance in the next task. The output variable “performance level” reveals how many symbols were assigned correctly and is interpreted as an indicator of the ability to concentrate on a task.

*Barrat Impulsiveness Scale—BIS-15* (Spinella, 2007, German translation from Meule et al., 2011): the BIS-15, a self-rating questionnaire which measures the construct of impulsivity, was added to the test-battery. It comprises 15 items that load on three independent factors: non-planning impulsivity (BISnp), motor impulsivity (BISm) and attentional impulsivity (BISa). The scale requires participants to estimate how much they agree with each statement on a 4-point Likert-type scale (1 = rarely/never, 4 = almost always). We included this test because BCI users who had higher scores on the BIS-11 (the BIS 15 is a short version of the BIS-11) had lower P300 amplitudes in a conventional oddball task (Russo et al., 2008).

#### Clinical tests

*Allgemeine Depressionsskala—ADS-L* (Hautzinger and Bailer, 1993): the ADS-L is the German version of the Center for Epidemiologic Studies Depression Scale (Radloff, 1977). It is a self-report depression scale designed for the general population.

To measure the subjects’ current mood during the BCI session, we applied the subscale “current mood” of the “*Skalen zur Erfassung der Lebensqualität”* (SEL, English: scales to assess quality of life; Averbeck et al., 1997). Participants’ current motivation just before the BCI session was assessed with an adapted version of the “*Questionnaire for Current Motivation”* (QCM; Rheinberg et al., 2001; Nijboer et al., 2008) which comprises 18 statements to be rated on a 7-point Likert-type scale and load on four sub-scales (“mastery of confidence”, “fear of incompetence”, “interest” and “challenge”). Mood and motivation data were reported elsewhere (Kleih et al., 2013).

### Experimental BCI setup

During the SMR-BCI sessions, EEG was acquired from 16 passive Ag/AgCl electrodes, mounted into a 64-channel cap (*Easycap GmbH, Germany*) at positions (FP1, FP2, F3, Fz, F4, T7, C3, Cz, C4, T8, CP3, CP4, P3, Pz, P4, Oz). Ground and reference electrodes were placed at the mastoids. Signals were amplified with a 16-channel g.USBamp amplifier (*g.tec Medical Engeneering GmbH, Austria*) and recorded at a sampling rate of 256 Hz with online 50 Hz-notch filter using the BCI2000 software (Schalk et al., 2004). For calibration measurement the stimulus presentation module was used, the classic cursor task paradigm was used for the feedback sessions, both were implemented in BCI2000. The analysis of the calibration data was conducted with the Offline Analysis tool of BCI2000.

For each electrode, we computed the power spectrum in the range 0–40 Hz to identify the determination coefficients between conditions in the alpha and beta bands. For cursor feedback, we chose those two of the three possible combinations (rH vs. lH, rH vs. F, lH vs. F), that displayed highest determination coefficients and selected one electrode per combination. Activity from this electrode at a given frequency band was used to control the cursor during cursor feedback using a linear discriminant analysis classifier.

### Data processing

All data processing was performed with MATLAB 2010b (*The Mathworks, USA*) except for statistical analysis which was calculated with SPSS 18.0 (*IBM, USA*).

According to Hammer et al. (2012), SMR-BCI performance equaled the percentage of correct responses, i.e., cursor movement according to the task requirements within one run and served as dependent variable in the further analyses. Training sessions consisted of 12 runs comprising 25 trials each.

### Statistical analysis

Normal distributions of data were checked with Kolmogorov-Smirnov tests and with visual inspection of the QQ-Plots. Either Pearson (when variables were normally distributed) or Spearman correlation coefficients (if variables were not normally distributed) were calculated between psychological parameters and SMR-BCI performance. For all analyses, the respective probability of type I error was maintained at the level of *α* = 0.05. For the psychological tests, we calculated percentile ranks (PR) if correspondent norms were available—if not, we used the cumulative values. To evaluate whether a learning progress could be observed across the three feedback sessions and whether the feedback paradigm influenced performance, a two-way repeated-measures ANOVA was conducted with session (3) as within subject factor and feedback paradigm (2) as between-subject factor.

To identify significant psychological predictors, we calculated logistic regression analyses. Since SMR-BCI performance was not distributed normally, we transformed the values according to the following function: Li = ln(Zi/(1−Zi)), where Zi denotes the SMR-BCI performance value of i on a scale from 0 to 1. To detect possible predictors, a variable selection procedure in each subgroup of psychological tests (performance, personality, clinical) was performed. In each test block, we searched for psychological variables which were significantly correlated with feedback performance, and at the same time were not inter-correlated in the same subgroup. To solve the problem of multiple comparisons, we corrected according to Bonferroni in each subgroup of tests.

All psychological variables that remained after the reduction procedure were included as independent variables into the regression model. To further investigate the validity of the two predictors described by Hammer et al. (2012), we predicted BCI performance achieved in the present study on the basis of the 2HAND values (overall mean error duration) and on the AHA values (performance level) which were obtained by Hammer et al. (2012). The prediction was based on the two regression models described by Hammer et al. (2012, see equations 1 and 2):

Model for visuo-motor control ability (2HAND, Hammer et al., 2012):

(1) Predicted Accuracy=0.301 × 2HAND + 61.06

Model for performance level (AHA, Hammer et al., 2012):

(2) Predicted Accuracy=0.457×AHA + 44.63

## Results

### SMR-BCI online performance

Mean SMR-BCI performance across all feedback sessions was *M* = 79.00% (*SD* = 11.1; range 55.41–92.41). In the first session mean performance was *M* = 75.6% (*N* = 32, *SD* = 14.10), in the second *M* = 75.8% (*N* = 32, *SD* = 15.02) and in the third *M* = 79.2% (*N* = 28, *SD* = 11.01). To test whether learning occurred and whether the feedback design significantly affected performance, a 3 × 2 repeated-measures ANOVA was conducted with time (sessions 1–3) as within and group (2) as between subject factors. We neither found an effect of time (*F*_2,52_ = 0.211; *p* = 0.811) nor of type of feedback (*F*_2,52_ = 0.880; *p* = 0.421) and no interaction.

Subjects (*N* = 4) who performed on chance level in sessions 1 and 2 were not invited for the third session to avoid frustration. Thus, for all further analyses we used the feedback performance of sessions 1 and 2 (*M* = 75.67, *SD* = 14.07). The criterion level of >70% (Kübler et al., 2001) was reached by 68.8% of participants (*n* = 22). No significant correlation between age and SMR feedback performance was found (Spearman’s rho = −0.17; *p* = 0.35).

### Predictor analyses

As expected, in the test category “performance tests”, the 2HAND variable “overall mean error duration” was moderately correlated with performance (*r* = 0.36; *p* < 0.05) but failed significance after Bonferroni correction (adjusted α-level *p* = 0.008). Furthermore, one variable of the subgroup “personality tests” was significantly correlated with SMR feedback performance i.e., “attentional impulsivity”, a subscale of the Barrat Impulsiveness Scale (BIS-15; *r* = −0.41; *p* < 0.05; adjusted α-level *p* = 0.0025) which also failed significance after Bonferroni correction. Unexpectedly, the variable “performance level” (AHA) was not significantly correlated with performance (*r* = 0.24; *p* = 0.195). None of the clinical tests predicted performance. “Attentional impulsivity” and “overall mean error duration” were moderately inter-correlated (*r* = −0.39; *p* < 0.05).

The regression of these two variables on BCI feedback performance explained almost 20% of the variance (*R*^2^ = 0.197; *F*_2,29_ = 3.55; *p* < 0.05). On its own “overall mean error duration” explained only 8% (*R*^2^ = 0.082; *F*_1,30_ = 2.69; *p* = 0.112) of the variance and was not significant. For the entire regression model, “attentional impulsivity” remained significant (*p* = 0.05), but “overall mean error duration” did not (*p* = 0.44). In a further step, we conducted robust regression analyses for the independent variables “overall mean error duration” and “attentional impulsivity” to monitor the influence of potential outlier values. We also performed this analysis for the variable AHA “performance level” to check whether outlier values were responsible for the low and non-significant correlation coefficient. In Table 1 the regression coefficients for standard regression models and for the robust regression analyses are presented. Least square regression models did not significantly differ from robust regression models in any of the predictor variables. We thus conclude that the models were robust with regards to outliers.

**Table 1:** **Regression coefficients for standard regression models and for robust regression analyses**.

	**Least square**	**RMS error**	**Robust regression**	**RMS error**	**Comparison of regression slopes**
2HAND	*Y* = 64.76 + 0.33*X	13.37	*Y* = 63.90 + 0.36*X	14.18	*t* = 0.1224, *p* > 0.05
BIS_A	*Y* = 103.01 + −2.89*X	13.04	*Y* = 108.98 + −3.43*X	13.44	*t* = −0.3240, *p* > 0.05
Performance level	*Y* = 64.76 + 0.15*X	14.00	*Y* = 63.76 + 0.18*X	15.19	*t* = 0.1046, *p* > 0.05

### Stability of the prediction model

Although the 2HAND failed significance in the entire regression analysis, the correlation with SMR feedback performance was at a similar level as in Hammer et al. (2012; *r* = 0.42). Consequently, we conducted a further analysis to verify the stability of the predictor models.

**Figure 2:**
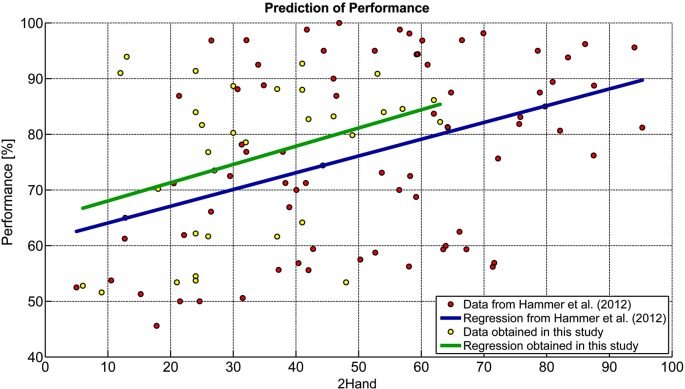
**Comparison of prediction models.** Data from Hammer et al. (2012) were compared to those obtained in this study.

Comparisons of the predictor analysis reported by Hammer et al. (2012) with the results obtained in this study are presented in Figure 2. Regression coefficients were not statistically different (*t* = −0.144, *p* = 0.885) between the studies, confirming the stability of the results reported in Hammer et al. (2012). From the model described by Hammer et al. (2012; Predicted Accuracy = 0.301 × 2HAND + 61.06) we could predict current performance with an average prediction error of *M* = 12.07% (*SD* = 6.67, range: 2.12–28.96%). In more than 50% of participants, prediction error was below 10% and in 75% it was below 15%. Predicted values significantly correlated with achieved performance (*r* = 0.36, *p* < 0.05). To further consolidate the prediction model we merged both data sets into a new regression model displayed in equation 3. This relationship was highly significant (*r* = 0.39, *p* < 0.0001).

(3) Predicted Accuracy=0.269×2HAND + 63.87

For the AHA “performance level” such analysis was not conducted because the correlation with SMR feedback performance was not significant.

## Discussion

The purpose of the current study was to investigate whether psychological variables could predict performance with a BCI based on modulation of SMR and whether we could validate our previously found predictors of such performance. To investigate solely potential predictors of human learning, no machine learning procedures were applied.

### Predictors found and comparisons with the literature

Contrary to our expectation, only few psychological variables were related to SMR-BCI performance, although we used an SMR-BCI approach that did not involve machine learning.

Visuo-motor coordination ability (here: mean error duration) and impulsivity were positively correlated with SMR feedback performance, but the ability to concentrate on a task (AHA), a previously found predictor, was not. The regression model on the basis of the current data identified impulsivity as the sole predictor explaining about 20% of the variance. However, as visuo-motor coordination ability correlated with performance, we applied the regression model described by Hammer et al. (2012) to the data. This rendered the 2HAND predictor again significant which held also true for the merged data set. This indicates that visuo-motor coordination ability may be a stable, albeit small predictor of SMR performance. Furthermore, above all differences between the BBCI and SMR-BCI, that was used in the current study, this result suggests, that both approaches require similar abilities, and have a similar neurophysiological basis.

The impact of visuo-coordination abilities on SMR feedback performance is in accordance with the idea that neurofeedback learning is similar to motor learning (Lang and Twentyman, 1976). Lang and Twentyman proposed that the ability to control one’s own heart rate could be conceptualized as the acquisition of motor learning. They stated that the same processes were necessary to achieve control over cardiovascular processes as well as to hit a tennis ball correctly. In line with this concept, individuals who had good visual-motor coordination abilities showed better performance in the current study. The BIS-15 subscale “attentional impulsivity” (BISa) measures the ability to focus attention or to concentrate. Users with high scores on this subscale have difficulties in focusing attention, especially in monotonous tasks. The BISa was negatively related with SMR-BCI performance. The psychological construct impulsivity reflects a human predisposition to show impulsive behavior and actions across diverse situations. Russo et al. (2008) already discussed the influence of impulsivity on the P300 amplitude. They stated that users who had higher impulsivity scores had lower P300 amplitudes in a conventional oddball task. The authors pointed out that “impulsivity exerts a disadvantageous influence on the performance of tasks in which exclusive concentration and sustained attention, combined with the suppression of other behavioral impulses, are necessary” (Russo et al., 2008, p. 116). The suppression of spontaneous actions and movements during a BCI session and the ability to sustain attention in a little stimulant environment (especially during the screening session when participants receive no feedback) are important preconditions for good performance also in the SMR-BCI.

This result is also in line with the assumption that performing a BCI task requires self-regulatory capacities to focus on and comply with the task despite distracting thoughts and other interferences. Halder et al. (2011) found that good BCI users activated the supplementary, premotor, and, importantly, prefrontal areas significantly stronger than users with worse results. Prefrontal areas, specifically the dorsolateral prefrontal cortex, are well known to be crucial for the allocation of attentional resources (Smith and Jonides, 1999).

However, this result is in contrast to the lack of correlation between the variable “performance level” of the AHA and BCI performance in the current study, since the “performance level” can be interpreted as an indicator of the ability to concentrate. Hammer et al. (2012) reported a correlation of *r* = 0.50 between SMR-BCI performance and the “performance level”; yet the current study displayed also a positive, but non-significant correlation of *r* = 0.24. Presumably, “attentional impulsivity” and “performance level” capture different components of attention. Further, both measures are assessed differently: while “attentional impulsivity” is estimated by means of self-report, “performance level” is the result of a performance test.

No other psychological variable was significantly correlated with performance. We refrained from applying any machine learning algorithms to ascribe any improvement of performance to human learning, but no learning occurred within the three sessions. This is actually an observation often reported in SMR-BCI performance. Some subjects have spontaneously access to the cortical activation patterns that lead to successful BCI control. However, to learn motor imagery based BCI control if such control is not spontaneously available and to improve the baseline level, it seems that longer training is necessary (Friedrich et al., 2009). In patients with ALS learning did not occur until after the 10th session and performance increased as a function of time (Kübler et al., 2005).

### Practical implications

Obviously, the practical value of the visuo-motor coordination ability for predicting later SMR-BCI performance is low because this predictor cannot be assessed and trained in potential BCI users in the locked-in state, in who no muscular movement is possible.

However, reliable and replicable predictors of BCI performance contribute to a better understanding of the correlates of BCI control. In their model of BCI control Kübler et al. identified four factors that influence BCI performance: the individual, i.e., psycho-biological variables; the technical, i.e., hard- and software components; the BCI paradigm, i.e., how many degrees of freedom, which instruction and modality; and the application, i.e., what effector (spelling, gaming etc.) is controlled by the BCI to interact with the environment (Kübler et al., 2011). Our results contribute to the first factor, i.e., the contribution of individual aspects, here psychological-behavioral, to BCI performance.

When further elucidating the correlates of BCI control it may, in the future, be possible to assign weights to the different factors dependent on the input signal for BCI control. Future development could then focus on these aspects specifically with targeted end-users of BCI.

To further strengthen (or reject) the impact of visuo-motor coordination ability for acquiring SMR-BCI control, subjects could be trained in visuo-motor skills prior to BCI sessions and the effect on later SMR-BCI performance could be measured.

To further elaborate “attentional impulsivity” as reliable predictor of SMR-BCI performance, users who present with high impulsivity scores could participate in an attention training prior to the BCI task. Such trainings are applied successfully to improve concentration in people with attention deficit disorders and after stroke and are more accessible than the visuo-motor tasks with reduced motor control (refs). To be of value for potential BCI end-user with disease, the predictors need to be confirmed in clinical samples, which is, however, difficult due to the required larger sample size.

### Limitations

Finally, some limitations of the current study must be mentioned: We recruited a sample of young and healthy people who had a high level of education. Such a selection of participants renders the sample more homogenous than the general population and leads to reduced variance (Hammer et al., 2012). In more representative samples or in samples of patients with diseases that affect the central nervous system (e.g., ALS, epilepsy, stroke) psychological factors may prove more influential. In relation to the large number of psychological test variables, the sample size was too small to maintain significance after Bonferroni correction. Furthermore, we only conducted three BCI sessions, which may not be sufficient for learning. Another limitation is the low correlation of the visuo-motor coordination ability and SMR-BCI performance and the lack of significance in the regression model in the current data set. However, when enlarging the data set the predictor could be consolidated.

## Conclusions

Psychological variables explain a moderate amount of the variance of SMR feedback performance. Visuo-motor coordination abilities could be consolidated as a small predictor of performance. Further studies with healthy people and end-users of BCI alike are necessary to consolidate or reject other variables contributing to BCI control, and to elaborate the model of BCI control (Kübler et al., 2011).

## Conflict of interest statement

The authors declare that the research was conducted in the absence of any commercial or financial relationships that could be construed as a potential conflict of interest.
